# Glucose-Regulated Protein 78 Interacts with Zika Virus Envelope Protein and Contributes to a Productive Infection

**DOI:** 10.3390/v12050524

**Published:** 2020-05-09

**Authors:** Jamie Royle, Carolina Ramírez-Santana, Snezhana Akpunarlieva, Claire L. Donald, Rommel J. Gestuveo, Juan-Manuel Anaya, Andres Merits, Richard Burchmore, Alain Kohl, Margus Varjak

**Affiliations:** 1 MRC-University of Glasgow Centre for Virus Research, Glasgow G61 1QH, UK; j.royle.2@research.gla.ac.uk (J.R.); claire.donald@glasgow.ac.uk (C.L.D.); r.gestuveo.1@research.gla.ac.uk (R.J.G.); 2 Center for Autoimmune Diseases Research-CREA, School of Medicine and Health Sciences, Universidad del Rosario, 110010 Bogotá, Colombia; cramirezsantana@gmail.com (C.R.-S.); anayajm@gmail.com (J.-M.A.); 3 Institute of Infection, Immunity and Inflammation, College of Medical, Veterinary and Life Sciences, University of Glasgow, Glasgow G12 8QQ, UK; snezhanaaaa@gmail.com (S.A.); richard.burchmore@glasgow.ac.uk (R.B.); 4 Division of Biological Sciences, College of Arts and Sciences, University of the Philippines Visayas, 5023 Miagao, Iloilo, Philippines; 5 Institute of Technology, University of Tartu, 50411 Tartu, Estonia; andres.merits@ut.ee

**Keywords:** Zika virus, proteomics, GRP78, virus–cell interactions

## Abstract

Zika virus (ZIKV; Flaviviridae) is a mosquito-borne flavivirus shown to cause fetal abnormalities collectively known as congenital Zika syndrome and Guillain-Barré syndrome in recent outbreaks. Currently, there is no specific treatment or vaccine available, and more effort is needed to identify cellular factors in the viral life cycle. Here, we investigated interactors of ZIKV envelope (E) protein by combining protein pull-down with mass spectrometry. We found that E interacts with the endoplasmic reticulum (ER) resident chaperone, glucose regulated protein 78 (GRP78). Although other flaviviruses are known to co-opt ER resident proteins, including GRP78, to enhance viral infectivity, the role ER proteins play during the ZIKV life cycle is yet to be elucidated. We showed that GRP78 levels increased during ZIKV infection and localised to sites coincident with ZIKV E staining. Depletion of GRP78 using specific siRNAs significantly reduced reporter-virus luciferase readings, viral protein synthesis, and viral titres. Additionally, GRP78 depletion reduced the ability of ZIKV to disrupt host cell translation and altered the localisation of viral replication factories, though there was no effect on viral RNA synthesis. In summary, we showed GRP78 is a vital host-factor during ZIKV infection, which may be involved in the coordination of viral replication factories.

## 1. Introduction

Zika virus (ZIKV) is a mosquito-borne virus of the Flaviviridae family, which was originally isolated from a Rhesus monkey in Uganda in 1947, and the first recorded human infection was identified there in 1962–1963 [1,2]. ZIKV has since emerged as an important human pathogen during the 2015–2016 outbreak across South and Central America [3]. ZIKV has been linked to severe neurological and immunological diseases, including a pattern of birth and developmental defects known collectively as congenital Zika syndrome and Guillain-Barré syndrome [4,5,6,7]. Despite this, there are currently no specific treatments for ZIKV infection available. As such, there is a pressing need to delineate virus–host interactions more fully to understand the viral life cycle and enable targeted approaches to therapy discovery [8,9].

ZIKV has a single-stranded, positive sense RNA genome approximately 11 kb in length, which is translated into a single polyprotein [8]. Processing by host and viral proteases liberates both structural (capsid (C), pre-membrane (prM), and envelope (E)) and non-structural proteins (NS1, NS2A, NS2B, NS3, NS4A, NS4B, and NS5) [10]. As with other flaviviruses, ZIKV entry into host cells is initiated by receptor-mediated endocytosis and is followed by trafficking through endosomes and the subsequent release of the genome into the cytoplasm [11,12,13]. Replication is tightly linked to virus induced re-modelling of the endoplasmic reticulum (ER) to form structures known as replication factories (RF) [14]. Following translation, progeny virions assemble in the ER prior to maturation in the trans-Golgi network and are subsequently exported from the cell [11,15]. Although some interactions between ZIKV non-structural proteins as well as C with host cell proteins have been described [16,17,18,19,20], knowledge about the functions/interactions of structural proteins during the viral life cycle is mostly lacking. Recently, it was shown that E protein of dengue virus (DENV) interacts with heat shock protein 90, with a decrease in the latter resulting in less cellular E protein and increased viral titres [21]. Other than this study, relatively little is known about intracellular interactors of E.

Structural analysis has revealed that 90 E dimers form an icosahedral scaffold that coats the mature ZIKV particle, comparable to related flaviviruses such as DENV [22,23]. While the role E plays in the life cycle as a whole has yet to be fully elucidated, it is known to be critical during viral entry and assembly [12]. Differences in glycosylation of ZIKV E can modulate virus infectivity, as shown in both mice and mosquito models. Lack of ZIKV E glycosylation reduced viremia in mice models, as it is likely needed to aid binding to lectin-expressing cells; however, prevention of glycosylation did not affect ZIKV neurovirulence. In addition, glycosylation was required for efficient transmission to *Aedes aegypti* mosquitoes [24,25]. Following internalisation, pH changes in the endocytic pathway induce structural changes in E that allow virus and host membrane fusion and genome release into the cytoplasm [15]. Subsequent interactions between the host cell and ZIKV E are not well understood. 

Here, we aimed to identify cellular protein interactors of ZIKV E during the viral life cycle to increase our understanding of E roles and functions during the viral life cycle. For this, we used a mass-spectrometry based proteomics approach, which we believed would give us the best overview of cellular proteins that interact with E and allow us to investigate the role and the relevance of some of those interactions further. From the data obtained, we identified an interaction between E and glucose-regulated protein 78 kDa (GRP78)—an essential protein for mediating the unfolded protein response (UPR) as well as an important ER-resident chaperone [26,27,28]. UPR proteins, including GRP78, may be important during ZIKV infection. Activation of this pathway has been linked to clinical features of ZIKV infection such as microcephaly [29]. Additionally, ZIKV has been shown to upregulate the production of UPR proteins, including GRP78, during infection of neural cell culture [30]. In this study, GRP78 was found to re-localise to sites of ZIKV E staining, and GRP78 expression was seen to increase after a 24 h infection. While chemical modulators of GRP78-mediated ER stress responses did not affect ZIKV replication, GRP78 depletion significantly reduced the production of infectious virus particles. Further experiments revealed that GRP78 is important for ZIKV replication post-entry but prior to maturation and egress. Depletion of GRP78 reduced viral protein synthesis but not viral RNA synthesis, and GRP78 is required for maintaining the ER localisation of viral RFs. This suggests a novel and important role for GRP78 in the ZIKV life cycle.

## 2. Materials and Methods

### 2.1. Virus Strains

*ZIKV/H. sapiens/Brazil/PE243/2015* (abbreviated to ZIKV PE243) used in the study was characterised and is available from the authors [31]. ZIKV PE243 was obtained from collaborators at passage 2, and a passage 3 working stock was expanded from this. The generation of a ZIKV nanoluciferase-expressing reporter virus in Vero E6 cells, here termed ZIKV-Nanoluc, has been described previously [32,33]. 

### 2.2. Cells

Human A549 (ECACC, UK, 86012804) and African green monkey Vero E6 (ATCC, Manassas, VA, USA, CCL-81™) cells were maintained in Dulbecco’s Modified Eagle’s Medium (DMEM, Thermo Fisher Scientific, Waltham, MA, USA) supplemented with 10% fetal bovine serum (FBS). A549 cells expressing bovine viral diarrhea virus (BVDV) *N*-terminal protease (NPro), abbreviated A549-NPro cells (kindly provided by R. E. Randall, University of St Andrews, St. Andrews, UK), were maintained as described as above but with the addition of 2 µg/mL blasticidin to select for cells expressing BVDV-NPro [34]. BVDV NPro has been shown to inhibit IRF3 signalling and therefore reduce type 1 interferon induction. These cells were used for amplifying ZIKV stocks and performing plaque assays where indicated [34]. All cell lines were incubated in a humidified 5% CO_2_ incubator at 37 °C. All live cell-culture work was conducted in biosafety cabinets. 

### 2.3. Virus Growth and Titration

Vero E6 cells were used to rescue ZIKV Nanoluc as previously described [33]. A549-NPro cells were infected with ZIKV PE243 or ZIKV-Nanoluc, and the supernatant was harvested following the observation of cytopathic effects (CPE) 5–7 days later. Supernatants were serially diluted onto A549-NPro cells and overlaid with DMEM supplemented with 2% FBS and 1.2% Avicel (FMC BioPolymer, Philadelphia, PA, USA) for 5 days at 37 °C. Cell monolayers were fixed with 4% formaldehyde for 20 min prior to staining with toluidine blue (Sigma-Aldrich, St. Louis, MO, USA) to visualise plaque formation. All infections were performed in DMEM supplemented with 2% FBS at 37 °C with 5% CO_2_.

### 2.4. Small Molecule Inhibitor Assays

Epigallocatechin-gallate (EGCG, Sigma-Aldrich, St. Louis, MO, USA, E4143) and honokiol (HNK, Sigma-Aldrich, St. Louis, MO, USA, H4914) were dissolved in DMSO at 100 mM and 10 mM, respectively, and stored at −20 °C. To determine cell viability, EGCG and HNK were serially diluted onto 1 × 10^4^ A549 cells for 26 h. CellTitre-Glo Luminescent Cell Viability Assay (Promega, Madison, WI, USA) reagent was then added to cells following manufacturer’s instructions. Values were normalised to DMSO controls. For infection assays, A549 cells were treated with EGCG or HNK 2 h prior to and/or throughout a 24 h infection with ZIKV-Nanoluc (multiplicity of infection (MOI) 0.1). Where specified, ZIKV-Nanoluc was incubated with either EGCG or DMSO for 2 h in DMEM plus 2% FBS prior to infection of cells. To measure activity of Nanoluc expressed by a virus, cells were first lysed in Passive Lysis Buffer (Promega, Madison, WI, USA) before detection with Nano-Glo Luciferase Assay System plate reader (Promega, Madison, WI, USA). Values were normalised to infected cells treated with DMSO only. All drug treatments were performed in DMEM supplemented with 2% FBS at 37 °C with 5% CO_2_. 

### 2.5. Transfection of Nucleic Acids and Silencing of Gene Expression

To knockdown GRP78 expression, A549 cells were seeded in 24-well plates at a density of 1 × 10^5^ cells per well before transfection of homogenous siRNA molecules targeting GRP78 (siG, Thermo Fisher Scientific, Waltham, MA, USA, s6980; siG #2, s6981). As a negative control, Neg control siRNA 2 (siN, Thermo Fisher Scientific, Waltham, MA, USA, 4390846) was used. When transfecting, 5 pmol of siRNA was used with 2 µL of DharmaFECT 2 (Horizon Discovery, Cambridge, UK) per well according to the manufacturer’s protocol. 

### 2.6. Freeze/Thaw Assay

A549 cells were treated with siN or siG and then infected with ZIKV PE243 at MOI 5 for 24 h. Supernatant was harvested from siN and siG cells and split into two fractions. The first fraction was serially diluted and titred on A549-NPro cells. The second was subject to 3× freeze/thaw (f/t) cycles, whereby supernatants were frozen on dry ice for 5 min and thawed at 37 °C for 2 min and titred on A549-NPros. Cell monolayers from corresponding cell supernatants were then washed with 1× trypsin and with 3× PBS washes to remove residual virus particles. These cells were subject to 3× f/t cycles, and cellular debris was pelleted at 4000× *g* for 10 min and clarified supernatant serially diluted onto A549-NPros to calculate titre.

### 2.7. Dual Luciferase Assay

A549 cells were treated with siN or siG for 72 h. Cells were infected with ZIKV-Nanoluc (MOI 5) for 48 h. For the final 24 h of infection, 100 ng of the firefly luciferase plasmid expression pGL4.13 (Promega, Madison, WI, USA) was transfected into cells using LT-1 Transfection Reagent (Mirus Bio, Madison, WI, USA) following manufacturers’ instructions. The Firefly luciferase gene in pGL4.13 is under the control of a cytomegalovirus (CMV) promoter, and its expression was used as a proxy for host-controlled translation. Cells were harvested and luciferase values were measured using the Nano-Glo Dual-Luciferase Reporter Assay System (Promega, Madison, WI, USA) following the manufacturers protocol.

### 2.8. Protein Immunoprecipitation (IP)

At 24 h post-infection (hpi), cells were scraped and washed with PBS before resuspension in lysis buffer (150 mM NaCl, 5 mM MgCl2, 20 mM HEPES (pH 7.4), 0.5% Triton X-100, 1:100 Halt protease inhibitor cocktail (Thermo Fisher Scientific, Waltham, MA, USA)). After lysis, 1/50 of sample was taken for immunoblot analysis and mixed with 4X Bolt LDS Sample Buffer (final 1× in H_2_O, Thermo Fisher Scientific, Waltham, MA, USA) and 10× Bolt Sample Reducing Agent (final 1× in H_2_O, Thermo Fisher Scientific, Waltham, MA, USA). Immunoprecipitation samples were kept on ice for 20 min, followed by centrifugation at 15,000× *g* at 4 °C for 20 min. The supernatant was transferred into fresh tubes on ice and incubated with mouse anti-ZIKV E antibody (Aalto Bio Reagents, Dublin, Republic of Ireland, AZ 1176) or rabbit anti-GRP78 antibody (Abcam, Cambridge, UK, ab21685) for 2 h at 4 °C. Following this, protein G magnetic beads (Dynabead Protein G, Thermo Fisher Scientific, Waltham, MA, USA) equilibrated with cold washing buffer (150 mM NaCl, 5 mM MgCl_2_, 20 mM HEPES (pH 7.4), 0.5% Triton X-100) were added for 1 h at 4 °C before 4× washes with cold washing buffer. Beads were finally re-suspended in sample buffer (1 Bolt LDS Sample Buffer, 1× Bolt Sample Reducing Agent in H_2_O) and subjected to proteomic analysis and/or Western blot. Before proteomic analysis, proteins were first digested with trypsin using the filter aided sample preparation (FASP) protocol as previously described [35].

### 2.9. Peptide Analysis by LC-MS/MS

Peptides were solubilised in 1% acetonitrile (ACN) (Rathburn Chemicals, Walkerburn, UK) with 0.05% formic acid (FA) (Sigma-Aldrich, St. Louis, MO, USA) and separated using an UltiMate 3000 RSLCnano liquid chromatography system (Thermo Fisher Scientific, Waltham, MA, USA) prior to online analysis by electrospray ionisation (ESI) mass spectrometry on an LTQ-Orbitrap Elite mass spectrometer (Thermo Fisher Scientific, Waltham, MA, USA). Peptide samples were desalted and concentrated for 4 min on a C18 trap column (5 mM × 300 µM ID, 5 µM, 100 Å) (Thermo Fisher Scientific, Waltham, MA, USA), washed for 7 min with 1% ACN with 0.05% FA at a flow rate of 25 µL/min, then separated through an Acclaim PepMap 100 C18 Column (150 mm × 75 µm ID, 3 µm, 100 Å) (Thermo Fisher Scientific, Waltham, MA, USA). The gradient, at a flow rate of 300 nL/min, was 4–40% of 80% ACN in 0.08% FA over 90 min, then 40–100% of 80% ACN in 0.08% FA over 14 min, held at 100% for 5 min, and then re-equilibrated to 4% of 80% ACN in 0.08% FA for a total of 125 min. Peptide ions were detected in the Orbitrap mass spectrometer with a precursor scan at 60,000 resolving power within the mass range of *m*/*z* 400–2000. Tandem mass spectrometry (MS/MS) was performed on the 20 most intense ions detected in each precursor scan, with singly-charged ions being excluded from the selection. MS/MS by collision-induced dissociation (CID) was carried out and detected in the linear ion trap (LTQ). A dynamic exclusion of 180 s was used to prevent repeated analyses of high intensity ions at the expense of lower intensity ions. 

### 2.10. Data Analysis

Raw data were analysed by Proteome Discoverer (v.2.1.1.21, Thermo Fisher Scientific, Waltham, MA, USA). Protein identification was performed against ZIKV PE243 in the UniProt/SwissProt database. Carbamidomethyl (C) was set as a fixed modification, while Oxidation (M) was set as a variable modification. The precursor and the fragment mass tolerances were set to 10 ppm and 0.6 Da, respectively, with up to 2 missed cleavages allowed. The false discovery rate (FDR) was 0.01. 

### 2.11. Immunoblot Analysis

Proteins were separated on Bolt 4–12% Bis-Tris Plus gels (Thermo Fisher Scientific, Waltham, MA, USA) and transferred to Protran 0.45 NC membranes (GE Healthcare Life Sciences, Chicago, IL, USA) using the Trans-Blot SD semi-dry transfer cell. The membrane was blocked for 1 h in 5% *w*/*v* non-fat dry milk in 0.1% Tween-PBS. The membrane was then incubated overnight at 4 °C in 5% *w*/*v* non-fat dry milk in Tween-PBS containing either mouse anti-E (Aalto Bio Reagents, Dublin, Republic of Ireland, AZ 1176), rabbit anti-actin (Sigma-Aldrich, St. Louis, MO, USA, A2066), rabbit anti-NS5 [33], rabbit anti-gamma tubulin (Sigma-Aldrich, St. Louis, MO, USA, T3559), or rabbit anti-GRP78 (Abcam, Cambridge, UK, ab21685). Following 3 × 10 min washes with Tween-PBS, the membranes were then incubated for 1 h in 5% *w*/*v* non-fat dry milk in Tween-PBS with anti-mouse or anti-rabbit secondary antibody conjugated with either horseradish peroxidase (HRP) (Abcam, Cambridge, UK) or a near-infrared fluorescent dye (LI-COR Biosciences, Lincoln, NE, USA). This was followed by three 10 min washes with 0.1% Tween-PBS buffer; enhanced chemi-luminescence reagent (Thermo Fisher Scientific, Waltham, MA, USA) was used for signal detection of HRP antibodies, whereas fluorescently labelled antibodies were imaged on an Odyssey CLx (LI-COR Biosciences, Lincoln, NE, USA). 

### 2.12. Immunofluorescence Microscopy

A549 cells were seeded at a density of 5 × 10^4^ cells per well onto 13 mm coverslips and allowed to settle overnight. Cells were infected with ZIKV PE243 (MOI 1) for indicated durations and fixed with 4% formaldehyde for 20 min at room temperature. Cells were then permeabilised with 0.5% Triton X-100 in PBS for 10 min and blocked with 5% FBS in PBS for 1 h. Antibodies targeting ZIKV E (mouse) or GRP78 (rabbit) or dsRNA (mouse, J2, Scicons, Budapest, Hungary) were diluted 1:1000 in 5% FBS in PBS and incubated with cells for 2 h at room temperature. Following this, staining with AlexaFluor secondary antibodies (Thermo Fisher Scientific, Waltham, MA, USA) diluted 1:1000 in 5% FBS in PBS was done for 1 h. Coverslips were mounted onto microscope slides using HardSet Antifade Mounting Medium with 4′,6-diamidino-2-phenylindole (DAPI) (VectorLaboratories, Burlingame, CA, USA). Images were taken on a Zeiss LSM 710 inverted confocal microscope (Carl Zeiss, Jena, Germany).

### 2.13. cDNA Synthesis and RT-qPCR

Trizol (Thermo Fisher Scientific, Waltham, MA, USA) was added to 2 × 10^5^ A549 cells per well in a 24-well plate with triplicate wells pooled to isolate total RNA and purified according to the manufacturer’s instructions. For cDNA synthesis, 1 µg RNA was used as template for Superscript III reverse transcriptase (Thermo Fisher Scientific, Waltham, MA, USA) with random primers (Promega, Madison, WI, USA). Quantitative RT-PCR for ZIKV and the housekeeping gene, GAPDH, was performed using specific primers (ZIKV: Fwd: GTTGTCGCTGCTGAAATGGA Rev: GGGGACTCTGATTGGCTGTA GAPDH: Fwd: GGTGGTCCAGGGTTTCTTA Rev: GTTGTCTCCTGCGACTTCA). Signal was detected using SYBR green Mastermix (Thermo Fisher Scientific, Waltham, MA, USA) and an ABI7500 Fast qPCR machine according to manufacturer’s protocol. Results were analysed using the ΔΔCt method [36]. 

### 2.14. Statistical Analysis

Data were visualised and analysed in GraphPad Prism v.9 (San Diego, CA, USA). Statistical significance was assessed using two-tailed unpaired Student’s *t*-test with Welch’s correction.

### 2.15. Data Availability

Data used to compile all figures found in this paper can be located here: http://dx.doi.org/10.5525/gla.researchdata.959.

## 3. Results

### 3.1. ZIKV E Interacts with GRP78

To identify interactors of ZIKV E, A549 cells were mock-treated or infected with ZIKV PE243 before cells were harvested, lysed, and immunoprecipitated using a ZIKV E-specific antibody. Samples were subjected to proteomic analysis, and multiple putative protein partners were identified (Table 1). A protein was considered a potential interactor if it was absent in at least two of the control samples but present in at least two replicates from infected cells. A full list of identified proteins is recorded in Supplementary Table S1. Proteins identified included glucose-regulated protein 78 kDa (GRP78, also known as HSPA5 or BiP), an ER-resident protein chaperone involved with coordinating cellular stress responses [27,37]. As GRP78 has been shown to be important in a range of other viruses, including other flaviviruses, it was chosen for further investigation here [38,39,40].

**Figure 1:**
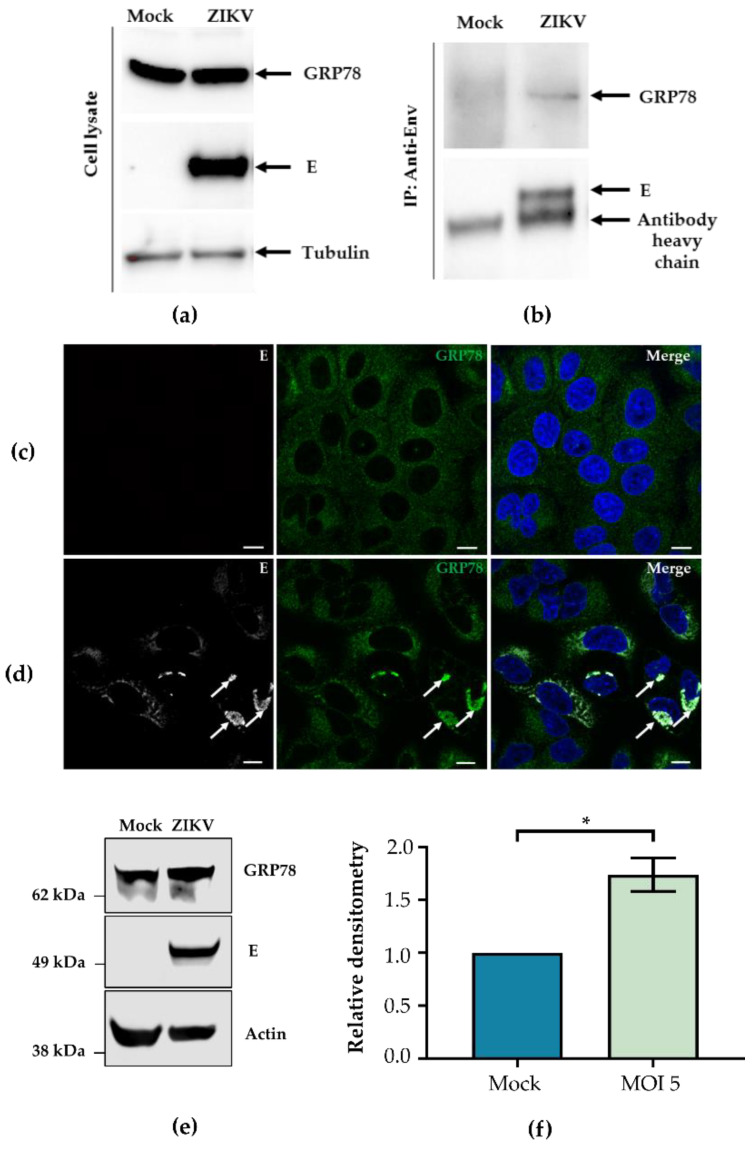
ZIKV infection modulates GRP78 localisation and expression in A549 cells. A549 cells were infected with ZIKV PE243 at multiplicity of infection (MOI) 1 for 24 h. (**a**) Cells lysates were probed for protein expression by Western blot analysis with antibodies against GRP78, ZIKV E, and a tubulin loading control; (**b**) these cell lysates were incubated with a ZIKV anti-E antibody prior to the addition of protein G magnetic beads to carry out immunoprecipitation (IP). This was followed by Western blot analysis. A549 cells seeded onto coverslips were (**c**) mock treated or (**d**) infected with ZIKV PE243 at MOI 0.1 for 24 h. ZIKV E is shown in white, GRP78 in green, and nuclei were stained with DAPI shown in blue. Scale bars represent 10 µm. Images were taken on an LSM 710 confocal microscope. (**e**) A549 cells were infected with ZIKV PE243 at MOI 5 for 24 h. Western blots for GRP78, E, and actin expression were performed on cell lysates. A representative image is shown. (**f**) Densitometry of GRP78 expression from triplicate Western blots shown relative to an actin loading control. Error bars represent standard error of the mean of triplicate repeats. An unpaired Student’s *t*-test with Welch’s correction was used to determine statistical significance where * *p*-value < 0.05. (**a**–**e**) Shown are representative images from triplicate experiments.

The interaction between GRP78 and ZIKV E was confirmed by co-IP with a ZIKV E-specific antibody. Cell lysates were analysed for GRP78, E, and tubulin expression (Figure 1a); the same lysates were subjected to IP using an anti-E antibody, and samples were probed for GRP78 and E (Figure 1b). GRP78 and E were also shown to co-localise using immunofluorescence staining. In mock-infected A549 cells, GRP78 displays a diffuse localisation (Figure 1c). However, following infection, GRP78 was seen to localise to a perinuclear location coincident with sites of E staining (Figure 1d). To investigate the expression of GRP78 following infection, lysates of mock or ZIKV PE243 infected A549 cells were harvested and analysed via Western blot for GPR78; a representative blot is shown (Figure 1e). Densitometry of triplicate Western blots revealed a significant increase of GRP78 levels relative to an actin loading control (Figure 1f), if cells infected at MOI of 5.

### 3.2. Small Molecule Inhibitors of GRP78-Mediated UPR do not Affect Viral Replication

**Figure 2:**
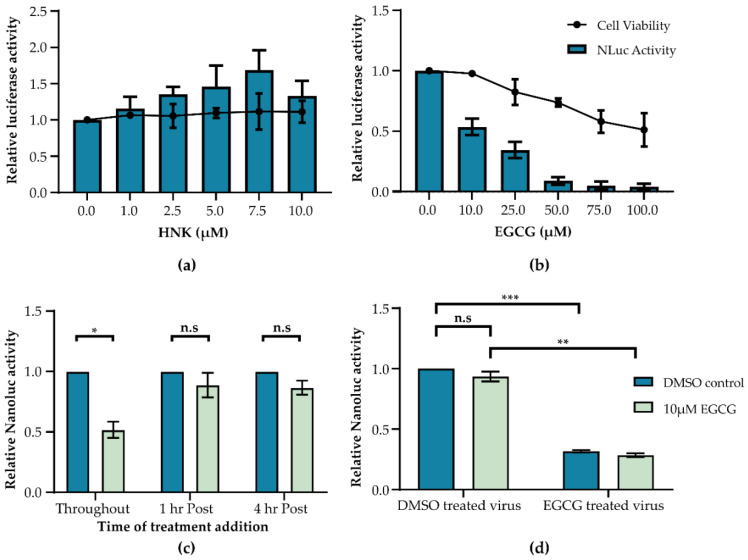
EGCG inhibits ZIKV independent of GRP78. A549 cells were treated with either (**a**) honokiol (HNK) or (**b**) epigallocatechin-gallate (EGCG) at indicated concentrations for 26 h. Cell viability was measured relative to a DMSO control as indicated by the line graph. Separately, A549 cells were treated with the same drug concentrations for 2 h prior to infection with ZIKV-Nanoluc (MOI 0.1) for 24 h. Nanoluc readings are relative to a DMSO control indicated by the bar graph. (**c**) EGCG (10 µM) was added to A549 cells and maintained throughout a 24 h ZIKV-Nanoluc infection or added 1 h or 4 h post-removal of virus inoculum. Nanoluc values were measured relative to a DMSO vehicle control. (**d**) ZIKV-Nanoluc was incubated with either DMSO or EGCG (10 µM) independently of cells for 2 h at 37 °C. Simultaneously, A549 cells were treated with DMSO or EGCG for 2 h before the drug containing media was replaced with drug treated or DMSO treated control virus for 1 h. Following this, virus inoculum was removed and replaced with DMEM containing either DMSO or EGCG as before for a further 23 h. Error bars represent the standard error of the mean of triplicate repeats. An unpaired Student’s *t*-test with Welch’s correction was used to determine statistical significance where n.s = not significant, * *p*-value < 0.05, ** *p*-value < 0.01 and *** *p*-value < 0.001. (**a**–**d**) *n* = 3.

Under non-stress conditions, GRP78 binds to and inactivates ATF6, IRE1, and PERK, three effector proteins of the UPR [41]. When exposed to stress stimuli, GRP78 undergoes a conformational change, leading to the release of effector proteins and activation of the UPR [42,43]. These effector proteins have myriad functions within the cell that aim to restore protein homeostasis or induce cell death if this is not possible [44]. Upon release from GRP78, PERK can phosphorylate the α subunit of the eIF2, leading to translational arrest, while IRE1 and ATF6 induce transcriptional upregulation of UPR members, resulting in a positive feedback loop [45,46,47]. The activity of the UPR can be blocked through the use of small molecule inhibitors such as (honokiol) HNK and (epigallocatechin-gallate) EGCG [48]. To test whether GRP78-coordinated stress responses impact ZIKV replication, A549 cells were treated with known inhibitors of GRP78, HNK (Figure 2a), or EGCG (Figure 2b) at indicated concentrations prior to and throughout infection with ZIKV-Nanoluc. The viability of cells in the presence of drugs alone was also plotted. HNK treatment had no effect on cell survival or Nanoluc readings at any concentration tested. In contrast, EGCG significantly reduced Nanoluc readings while having no effect on cell survival at 10 µM. To investigate this further, 10 µM EGCG was added to A549 cells at time points during or after ZIKV-Nanoluc infection and subsequently maintained throughout infection until cells were lysed and Nanoluc activity was observed (Figure 2c). Only when EGCG was in the supernatant at the time of infection was a reduction in Nanoluc readings observed. To establish whether GRP78 inhibition affected early stages of ZIKV infection or whether EGCG directly inhibited ZIKV binding to cells, ZIKV-Nanoluc was incubated with EGCG or a DMSO vehicle control prior to infection. When ZIKV-Nanoluc was incubated with EGCG before infection, Nanoluc readings were reduced regardless of whether cells had been pre-treated with EGCG or not (Figure 2d). In all cases, ZIKV Nanoluc readings were only reduced when EGCG encountered ZIKV in the inoculum, indicating EGCG could potentially act directly on virions by either inhibiting viral binding to the cell or through direct damage to viral particles. These data corroborate other results suggesting that EGCG can prevent viral fusion by binding to ZIKV E protein [49,50]. Together, these data show that inhibition of GRP78 using HNK and EGCG does not affect ZIKV infection in A549 cells.

### 3.3. GRP78 Depletion Reduces ZIKV Infectious Titre

**Figure 3:**
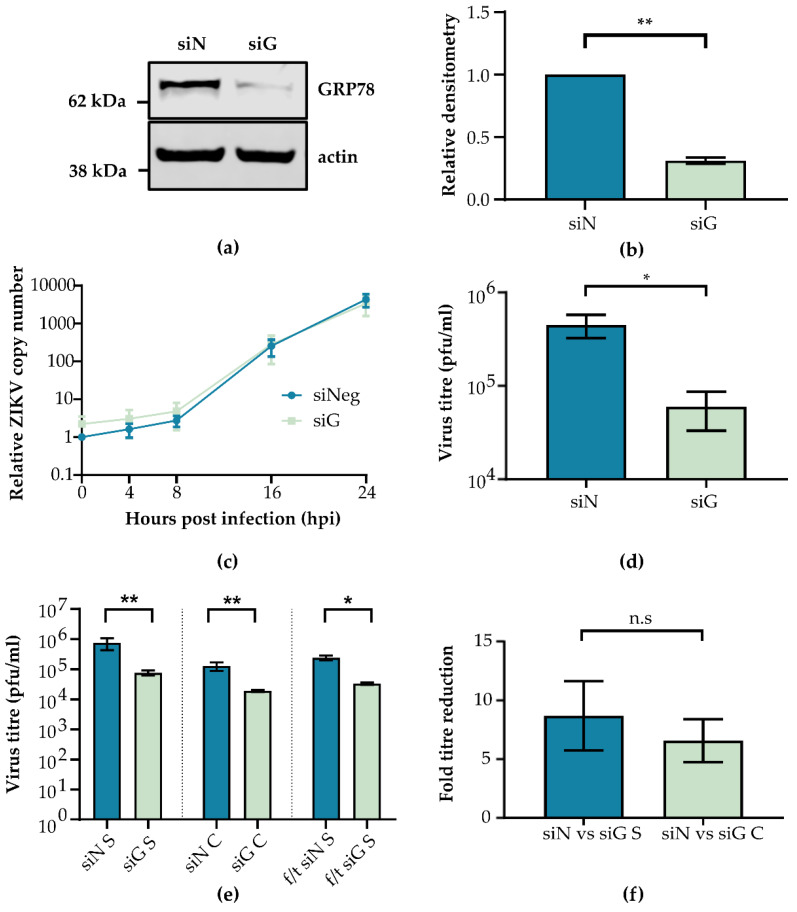
GRP78 depletion reduces ZIKV infectious particle release. A549 cells were treated with siRNA targeting GRP78 (siG) or negative control (siN) for 72 h. (**a**) A representative Western blot of triplicate repeats is shown. (**b**) Triplicate Western blots were analysed for GRP78 densitometry relative to actin levels to measure protein expression. (**c**) Cells treated with siRNA were infected with ZIKV PE243 at MOI 0.1. RNA was extracted at 4, 8, 16, and 24 h post infection. Viral RNA copy number was measured by RT-qPCR relative to a GAPDH control and normalised to viral RNA detected at 0 h in the siN sample. (**d**) Supernatant was harvested at 24 h post infection, and viral titre was measured via plaque assay. (**e**) Following siRNA treatment, cells were infected with ZIKV PE243 at MOI 5 for 24 h. Supernatant was harvested from siN and siG cells, and the titre was calculated for untreated supernatants (siN S and siG S) and freeze/thaw (f/t) supernatants (f/t siN S and f/t siG S). Monolayers corresponding to siN or siG supernatants were subject to 3× f/t cycles, and the titre was calculated (labelled siN C and siG C). (**f**) The reduction in titre between siN treated cells and siG shown in (**e**) is plotted, where siN vs. siG S represents samples taken from the supernatant, and siN vs. siG C is intracellular virus. The mean fold-reduction was calculated from three independent experiments, and error bars show the standard error of the mean. (**a**–**f**) Experiments were performed in triplicate, and the mean and the standard error of the mean are plotted. An unpaired Student’s *t*-test with Welch’s correction was used to determine statistical significance where n.s = not significant, * *p*-value < 0.05, ** *p*-value < 0.01.

Although inhibition of GRP78 mediated UPR responses appeared to have no effect on ZIKV replication, the importance of GRP78 itself during ZIKV infection was tested using GRP78-specific siRNA. GRP78 knockdown efficiency was assessed by comparing relative densitometry of GRP78 protein expression following siRNA treatment (siG) vs. control siRNA (siN). Western blots of cell lysates (Figure 3a) showed that siG treatment significantly decreased GRP78 expression compared to siN (Figure 3b). GRP78 knockdown cells were infected with ZIKV PE243, and total cellular RNA was isolated at regular intervals, while cell supernatant was harvested after 24 h. qPCR was performed on extracted RNA, and the fold change expression of viral RNA relative to a GAPDH control was calculated (Figure 3c). Cell supernatants were serially diluted onto A549-NPro cells, and plaque formation was determined (Figure 3d). GRP78 depletion did not appear to impact viral RNA production but did significantly reduce viral titres by ~7.5 fold.

To confirm the findings of siG, we tested another siRNA against GRP78 (siG #2), which similarly reduced GPR78 expression. Both siG and siG #2 reduced luciferase signal detection from ZIKV-Nanoluc infected cells (Figure S1). These data imply that GRP78 is important for ZIKV infection at a step downstream of genome replication. To further investigate whether viral entry to the cell is affected by GRP78 depletion, we probed for GRP78 protein expression on the cell surface. A549 cells appear to have low surface GRP78 expression compared to intracellular levels, and there was no significant change in ZIKV RNA levels following a high MOI infection in siN or siG treated cells (Figure S2).

As GRP78 depletion reduced ZIKV infectious particle release, and as GRP78 has been shown to be able to facilitate the egress and the trafficking of human cytomegalovirus, the possibility of an accumulation of intracellular ZIKV particles was investigated [40]. Intracellular viral particles were released from cells using multiple freeze/thaw (f/t) cycles as has been described previously [51]. To investigate this, A549 cells were treated with siN or siG and then infected with ZIKV PE243. Cell supernatants were harvested, split into two equal fractions, and titred by plaque assay. The first fraction compared viral titre between GRP78 depleted and control cells (Figure 3e, siN S vs. siG S), while the second fraction underwent 3× f/t cycles to control for the effect of f/t on viral titre (Figure 3e, f/t siN S vs. f/t siG S). Triplicate f/t cycles consistently reduced supernatant titre by ~5-fold. Subsequently, remaining cell monolayers were then washed to remove residual virus and subjected to 3× f/t cycles to liberate intracellular virus (Figure 3e, siN C vs. siG C). Importantly, there was no significant difference between the fold-reduction of supernatant (Figure 3f, siN vs. siG S) and intracellular (Figure 3f, siN vs. siG C) virus titres following siG treatment, indicating there was no accumulation of intracellular, infectious virions (Figure 3f). This suggested that GRP78 depletion does not block the export of infectious virus particles out of the cell and that the defect in virus production was before ZIKV egress.

### 3.4. GRP78 Knockdown Specifically Impacts Viral Translation 

**Figure 4:**
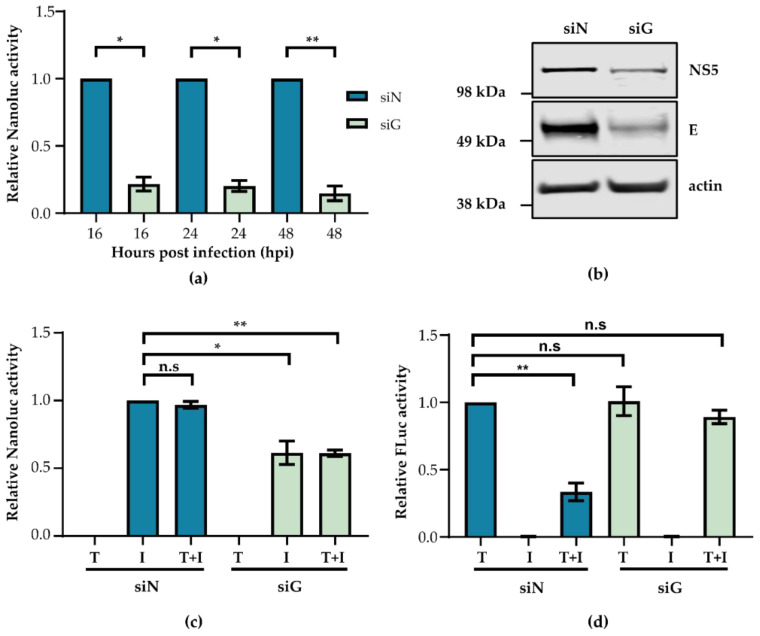
GRP78 depletion reduces ZIKV translation. A549 cells were treated with siN or siG for 72 h. (**a**) Cells were infected with ZIKV-Nanoluc at a MOI 0.1 before cells were lysed and Nanoluc levels were measured at 16, 24, or 48 hpi and normalised to each timepoint separately. (**b**) A549 cells were infected with ZIKV PE243 at MOI 10 for 24 h following siRNA treatment. A representative blot of triplicate experiments is shown detecting ZIKV NS5 and E with actin as loading control. (**c**,**d**) Following siRNA treatment, cells were either transfected with 100 ng of pGL4.13 FLuc expressing plasmid (labelled ‘T’), infected with Nanoluc at MOI 5 (labelled ‘I’) or both (labelled ‘T+I’). Cells were lysed, and (**c**) Nanoluc readings or (**d**) FLuc readings were measured. ZIKV-Nanoluc readings are relative to siN ‘I’, while FLuc readings are normalised to siN ‘T’. Experiments were performed in triplicate, and the mean and the standard error of the mean are plotted. An unpaired Student’s *t*-test with Welch’s correction was used to determine statistical significance where n.s = not significant, * *p*-value < 0.05, ** *p*-value < 0.01.

The previous results suggested that GRP78 was important during the ZIKV life cycle post- replication but pre-egress. To investigate whether viral protein translation and/or assembly were impacted by GRP78 depletion, A549 cells were treated with siRNA before infection with ZIKV-Nanoluc. Cells were lysed, and Nanoluc readings were measured (Figure 4a). At all time-points measured, there was a significant reduction in Nanoluc activity in siG treated cells. Additionally, translation of two ZIKV proteins, E and NS5, was found to be decreased in GRP78 depleted cells as measured by Western blot (Figure 4b). These results taken together with the data displayed in Figure 3 imply that GRP78 is required for viral protein translation.

Following this, we aimed to determine whether this decrease in viral protein production was because of a specific reduction in virus translation or a host-wide translation shut down. A549 cells were treated with siRNA and transfected with a pGL4.13 Firefly luciferase (FLuc) reporter plasmid to measure host translation in the presence or the absence of ZIKV-Nanoluc infection. Cells were lysed, and FLuc and Nanoluc readings were measured. Nanoluc readings were normalised to siN treated cells that had only been infected (I) (Figure 4c), while FLuc readings were normalised to siN treated cells that had only been transfected (T) (Figure 4d). As before, GRP78 knockdown significantly reduced Nanoluc readings (Figure 4c, siN I vs. siG I), while depletion of GRP78 in uninfected cells did not reduce FLuc production (Figure 4d siN T vs. siG T). This indicated that the reduction in ZIKV protein synthesis following GRP78 knockdown is specific to ZIKV protein production alone. Additionally, FLuc readings in siN treated and infected cells were significantly reduced, suggesting that ZIKV infection was hijacking or disrupting host translation machinery (Figure 4d, siN T vs. siN T+I). Surprisingly, ZIKV infection in siG treated cells did not reduce FLuc levels (Figure 4d, siG T vs. siG T+I), which suggests that GRP78 could be necessary to efficiently hijack cellular translational machinery, and this may be the mechanism by which GRP78 influences infection.

### 3.5. Viral dsRNA Localisation is Disrupted Following GRP78 Depletion

**Figure 5:**
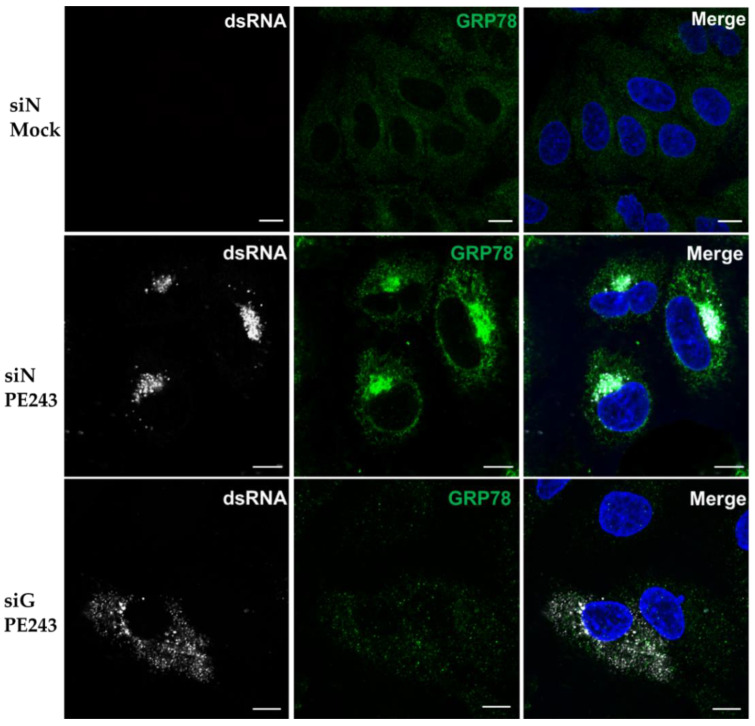
Viral dsRNA localisation is altered following GRP78 depletion. A549 cells were treated with siN or siG and mock infected or infected with ZIKV PE243 at MOI 0.1 for 24 h. Cells were stained with an anti-dsRNA antibody (white) and an anti-GRP78 antibody (green), and the nuclei were stained with DAPI (blue). Images were taken on an LSM 710 confocal microscope. Scale bars represent 10 µm, and images are representative of triplicate experiments.

To further explore whether GRP78 has a role in coordinating viral translation, the localisation of ZIKV double-stranded RNA (dsRNA) was investigated. dsRNA is an intermediate product of ZIKV replicative cycle and localises to RFs. These are hubs of viral replication and translation located on the ER [14,52]. A549 cells were treated with siN or siG and infected with ZIKV PE243. GRP78 and ZIKV dsRNA were then stained with specific antibodies (Figure 5). Mock infected cells showed no staining for dsRNA; however, in siN treated cells infected with ZIKV PE243, dsRNA clustered tightly to the perinuclear sites to which GRP78 re-localises during infection. As expected, treatment with siG significantly reduced GRP78 signal consistent with Western blot analysis (Figure 3a). In GRP78 depleted cells, dsRNA signal was diffuse throughout the cytoplasm in stark contrast to the highly localised staining seen in control cells.

## 4. Discussion

The emergence of ZIKV in the Americas highlighted the need for research to develop specific vaccines and therapeutics. In addition to viral proteins, host factors should be considered as candidates for treatments. Here, we analysed protein interactors of ZIKV E protein in human A549 cells and identified GRP78, a well-known ER-resident chaperone as a binding partner [53]. This interaction was verified by co-immunoprecipitation, and the co-localisation of E and GRP78 in cells was confirmed using confocal microscopy. Whether this interaction is direct or is mediated through other cellular or viral proteins has yet to be determined. It is possible active viral remodelling of cellular ER structures could result in proximity between GRP78 and E, giving rise to the observed interaction [14,54,55]. Another protein interactor of ZIKV E was 2′,5′-Oligoadenylate synthetase-like (OASL), an interferon stimulated gene that has been shown to be anti-viral against hepatitis C virus and has also been implicated in the response to ZIKV infection in placentas [56,57,58]. Information linking other protein interactors of ZIKV E identified in this study to other viruses is lacking; however, they may represent interesting targets for future studies.

Knockdown of GRP78 with specific siRNAs in A549 cells revealed it has a pro-viral role in ZIKV infection. GRP78 has previously been shown to enhance replication of other related flaviviruses. For example, GRP78 was reported to interact with Japanese encephalitis virus (JEV) E protein domain III and is an important factor facilitating virus entry into mouse neuronal cells, mouse primary neurons, and human hepatoma Huh7 cells [38]. Similarly, GRP78 is important for duck Tembusu virus entry [59]. Data regarding the role of GRP78 during DENV replication remains inconclusive. For DENV 2, it was found that GRP78 acts as an entry co-receptor in human liver HepG2 cells [60]. However, another study in HEK 293T cells concluded that GRP78 had no role in virus entry, although it was important during viral antigen production [39]. Similar to JEV, GRP78 interacted with DENV via domain III of DENV E protein [61]. The domain of ZIKV E that interacts with GRP78 has not yet been identified; however, it is possible they interact via domain III as seen for other flaviviruses. These data highlight that, while GRP78 seems to be a common co-factor for flaviviruses, the mechanism of action of GRP78 can be diverse.

To further characterise the pro-viral role of GRP78, we initially focused on the early stages of infection. GRP78 did not seem to be expressed at high levels on the surface of A549 cells, and GRP78 knockdown did not reduce incoming ZIKV copy number as measured by qPCR. Furthermore, GRP78 knockdown had no effect on viral RNA production over a time course of infection, together indicating that there was no defect in viral entry or replication. However, there was a significant reduction in the release of infectious viral particles. These results are in line with a previous study conducted with DENV, where depletion of GRP78 resulted in 100-fold reduction in virion production, but there was no effect on viral RNA synthesis [39]. Further experiments revealed that there was no defect in virus egress as a result of GRP78 knockdown cells and that ZIKV protein synthesis was reduced. Taken together, these data can explain the diminished production of new infectious virions, though we cannot exclude other explanations for this phenotype, including disruption of viral cap synthesis or protein processing and maturation. Interestingly, while viral protein synthesis was reduced, RNA synthesis was not affected. This could be explained by the fact that viral non-structural proteins have other roles aside from viral RNA replication, as shown by DENV and ZIKV NS5, the majority of which are located in the nucleus and not in RFs [62,63].

Reduced viral protein synthesis could have been linked to translational inhibition via the induction of ER stress. GRP78 is a well-known sensor of the UPR [53]. In non-stress conditions, GRP78 is bound to three effector molecules: IRE1, ATF6, and PERK. Cellular stress via the accumulation of unfolded proteins can cause the disassociation of these effector molecules from GRP78, leading to the activation of the UPR. One consequence is that PERK can phosphorylate eukaryotic translation initiation factor 2α, leading to the inhibition of cellular protein synthesis [64]. Our results showed GRP78 knockdown did not lead to a substantial decrease in cellular protein synthesis and that the decrease in ZIKV protein expression was specific to the virus. Whether the reduction in viral protein expression following GRP78 depletion was because of a reduction in viral specific translation or rather because of increased mis-folding and degradation on viral proteins has yet to be established. Additionally, two small molecule inhibitors of GRP78, HNK and EGCG, had no effect on virus replication once viral RNA had entered the cells. The anti-viral effect of EGCG was perhaps due to its ability to inhibit ZIKV virus entry, as was suggested previously, or potentially by damaging and inactivating viral particles [49,50]. HNK and EGCG bind the ATPase domain of GRP78 and maintain its unfolded conformation [42], thereby inhibiting binding of unfolded substrates and activation of the UPR [48]. It has not been determined if EGCG can interfere with the interaction between E and GRP78. The lack of a phenotype following drug treatment may indicate that UPR pathways do not affect ZIKV infection in A549 cells. This is in contrast to some studies that have linked ZIKV infection to the induction of the UPR in human fetal microcephalic cortices [29]. Here, the activation of the UPR is believed to impair corticogenesis and neurogenesis, a phenotype that was reversed with the addition of UPR inhibitors. Despite the difference in phenotype, it is worth noting that these observations were made in different model systems.

We also showed that, in siN-treated ZIKV infected cells, host cell translation was significantly reduced when compared to an uninfected control, a phenotype that has been reported for many viruses, including some flaviviruses [65,66]. It is interesting to note that, despite reduced cellular translation, GRP78 protein expression was maintained during ZIKV infection. Whether this was due to host stress response or modulated by the virus still needs to be determined. The mechanism by which ZIKV hijacks host translation has not yet been elucidated. Interestingly, in GRP78-depleted cells, we no longer saw a reduction in host cell translation. This perhaps indicated that ZIKV requires GRP78 to modulate host translation and could be the reason GRP78 depletion reduces ZIKV infection. The mechanism behind this phenotype remains unclear but could be related to the organisation and the localisation of viral replication factories. ZIKV replication factories are bound to ER membranes [14]. Similar to other arthropod-transmitted flaviviruses, such as DENV, West Nile virus, and tick-borne encephalitis virus [67,68,69,70,71], infection results in a complex redesign of the ER network. Such spatial organisation would likely allow for coordination of metabolites required for RNA replication and close proximity to the host cell’s translational machinery. Additionally, such complexes could protect viral RNA from detection by cellular anti-viral RNA sensors. All flavivirus proteins, including structural proteins, play a role in the formation of these structures [14,18,67,68,69,70,71,72,73,74]. dsRNA is formed at an intermediary step of ZIKV replication and can be used as a marker for virus replication complexes [14]. In control cells, ZIKV replication complexes clustered to a perinuclear locale as previously documented [14]. However, in GRP78 knockdown cells, dsRNA was more dispersed throughout the cell, indicating that GRP78 could be needed for coordination of replication complexes to areas that can support efficient viral protein synthesis, such as clustered to the ER. However, it is not yet clear how GRP78 can affect translation of viral RNA or how localisation and compartmentalisation of replication factories affect capturing of ribosomes and/or availability other resources.

It was proposed previously that GRP78 could represent a broad anti-viral target [75], as it has been shown to be pro-viral for viruses beyond the flavivirus genus, including chikungunya virus, Ebola virus, measles virus, influenza virus, hepatitis B virus, enterovirus, retroviruses, and coronaviruses such as Middle East respiratory virus [75,76,77,78,79,80,81,82,83,84]. GRP78 is a multi-faceted protein with diverse roles during viral life cycles; it has been shown to act as a (co-)receptor, to facilitate protein trafficking, and to function as a chaperone. As such, GRP78 is a promising target for the development of anti-viral drugs. The approach chosen highlights the strengths of proteomics approaches to understand virus–host interactions. Our findings suggest that ZIKV should be added to the growing list of viruses that depend on this important cellular factor, and the data show further roles for GFP78–E interactions that go beyond a role in entry. Indeed, our findings suggest that flaviviruses E proteins may use this cellular host factor in various ways, which points to broad and fundamental relevance of this protein–protein interaction.

## Figures and Tables

**Table 1:** Potential interactors of Zika virus (ZIKV) envelope (E) identified by proteomic analysis.

Protein	UniProt Accession	Infection, Sample 1	Infection, Sample 2	Infection, Sample 3	Control, Sample 4	Control, Sample 5	Control, Sample 6
LMNA	P02545	Yes	Yes	Yes	No	Yes	No
PGAM5	Q96HS1	Yes	Yes	No	Yes	No	No
GRP78	P11021	Yes	Yes	Yes	No	No	No
OASL	Q15646	Yes	Yes	No	No	No	No
TAO1	Q7L7X3	Yes	Yes	Yes	No	No	No

## Supplementary Materials

The following are available online at https://www.mdpi.com/1999-4915/12/5/524/s1, Figure S1: Uninfected A549 cells show no staining for dsRNA. Figure S2: GRP78 is not required for ZIKV entry. Table S1: Complete list of identified ZIKV E interactors.
